# Quantification of circulating endothelial and progenitor cells: comparison of quantitative PCR and four-channel flow cytometry

**DOI:** 10.1186/1756-0500-1-71

**Published:** 2008-08-28

**Authors:** Michael Steurer, Johann Kern, Matthias Zitt, Albert Amberger, Monika Bauer, Günther Gastl, Gerold Untergasser, Eberhard Gunsilius

**Affiliations:** 1 Tumor Biology and Angiogenesis Laboratory, Division of Hematology and Oncology, Innrain 66, Innsbruck Medical University, 6020 Innsbruck, Austria; 2 Tyrolean Cancer Research Institute, Innrain 66, 6020 Innsbruck, Austria

## Abstract

**Background:**

Circulating endothelial cells (CEC) and endothelial precursor cells (CEP) have been suggested as markers for angiogenesis in cancer. However, CEC/CEP represent a tiny and heterogeneous cell population, rendering a standardized monitoring in peripheral blood difficult. Thus, we investigated whether a PCR-based detection method of CEC/CEP might overcome the limitations of rare-event flow cytometry.

**Findings:**

To test the sensitivity of both assays endothelial colony forming cell clones (ECFC) and cord blood derived CD45^- ^CD34^+ ^progenitor cells were spiked into peripheral blood mononuclear cells (PBMNC) of healthy volunteers. Samples were analyzed for the expression of CD45, CD31, CD34, KDR or CD133 by 4-color flow cytometry and for the expression of CD34, CD133, KDR and CD144 by qPCR. Applying flow cytometry, spiked ECFC and progenitor cells were detectable at frequencies ≥ 0.01%, whereas by qPCR a detection limit of 0.001% was achievable. Furthermore, PBMNC from healthy controls (n = 30), patients with locally advanced rectal cancer (n = 20) and metastatic non-small cell lung cancer (NSCLC, n = 25) were analyzed. No increase of CEC/CEP was detectable by flow cytometry. Furthermore, only CD34 and KDR gene expression was significantly elevated in patients with metastatic NSCLC. However, both markers are not specific for endothelial cells.

**Conclusion:**

QPCR is more sensitive, but less specific than 4-channel flow cytometry for the detection of CEC/CEP cell types. However, both methods failed to reliably detect an increase of CEC/CEP in tumor patients. Thus, more specific CEC/CEP markers are needed to validate and improve the detection of these rare cell types by PCR-based assays.

## Findings

### CEC a heterogenous and rare cell type of the peripheral blood

**Figure 1:**
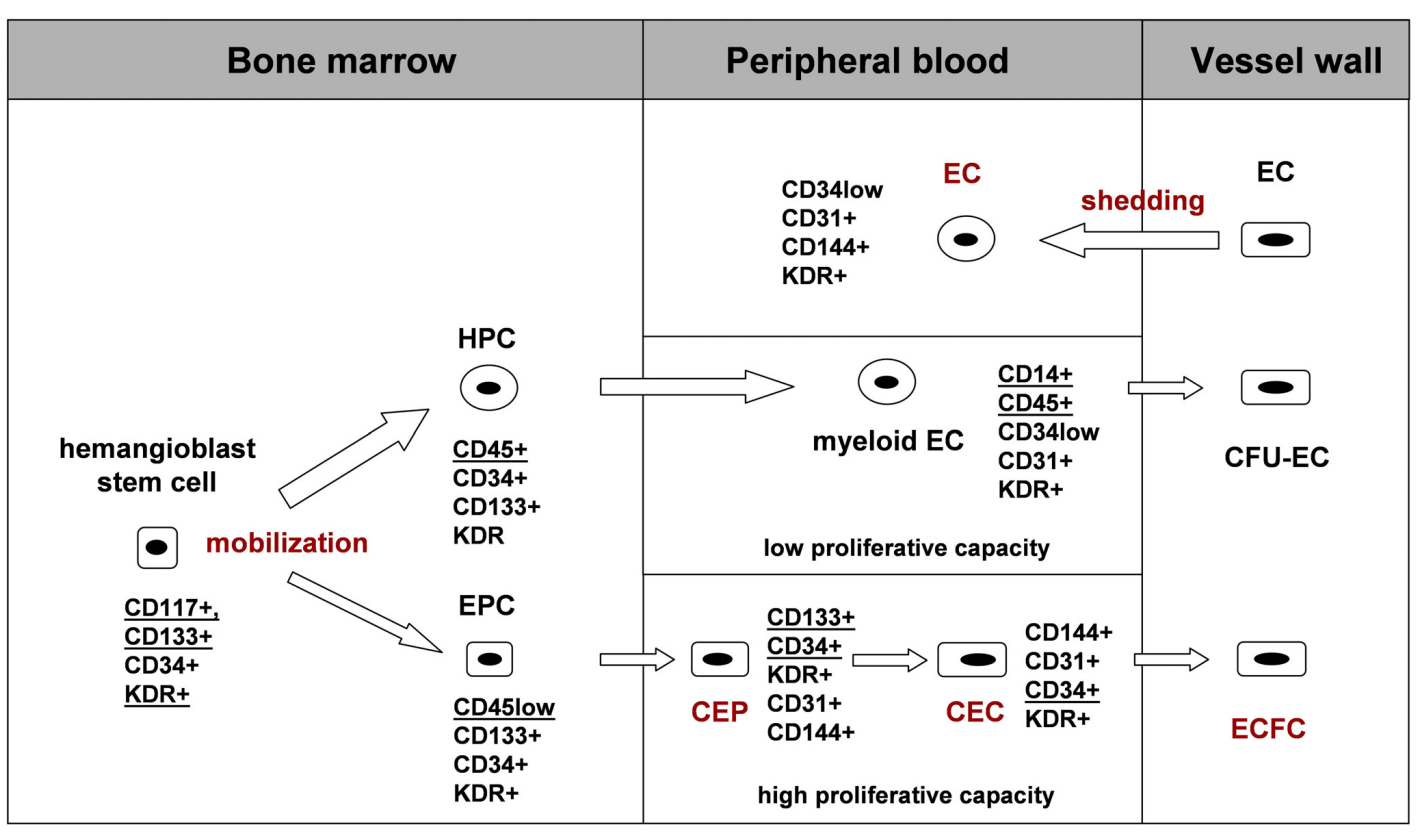
**Hypothetical model of the origin and immunophenotypic characteristics of distinct subpopulations of circulating endothelial cells in peripheral blood**. Pluripotent stem cells reside in the stem cell niche of the bone marrow and can give rise to "hemangioblasts" that have the capacity to differentiate into hematopoietic progenitor cells (HPC) or endothelial progenitors (EPC). EPC differentiate into circulating endothelial precursors (CEP) and circulating endothelial cells (CEC). HPC differentiate into myeloid cells such as monocytes, that can transdifferentiate into myeloid EC. Moreover, mature EC shed from the vessel wall can enter the circulation. Various subsets of circulating endothelial cell types have been demonstrated to contribute to tumor angiogenesis.

CEC have been shown to contribute significantly to angiogenesis in ischemia, inflammation, wound healing and tumor progression [1]. In cancer patients CEC measurement in peripheral blood has been proposed a non-invasive tool to assess tumor angiogenesis [2] and monitor antiangiogenic therapies [3] CEC comprise a heterogenous cell population consisting of endothelial cells shed from the vessel wall [4], bone marrow-derived CEP [5] and endothelial precursors originating from monocytic cells [6] (figure 1). CEC are only a tiny subset of the mononuclear cell fraction of peripheral blood rendering their quantification a challenging task. Endothelial colony formation assays are labor-intensive, time-consuming, poorly standardised and may give rise preferentially to monocytic cells [7]. Currently, the most common technique applied for CEC quantification is multicolor flow cytometry. Based on this method absolute numbers of CEC reported in the literature vary greatly ranging from 0 to 7.900 CEC/mL in peripheral blood of healthy controls and from 5 to 39.000 CEC/mL under pathological conditions [2,8]. Thus, there is an urgent need for more reliable and standardized methods for CEC quantification.

In this study both methods, flow cytometry and qPCR, were evaluated to compare the ability to detect mature peripheral blood-derived endothelial colony forming cells (ECFC; CEC phenotype) and cord blood-derived progenitors (CEP phenotype) spiked into PBMNC of healthy volunteers.

### Phenotype analysis of cord blood progenitor cells and ECFC by flow cytometry

**Figure 2:**
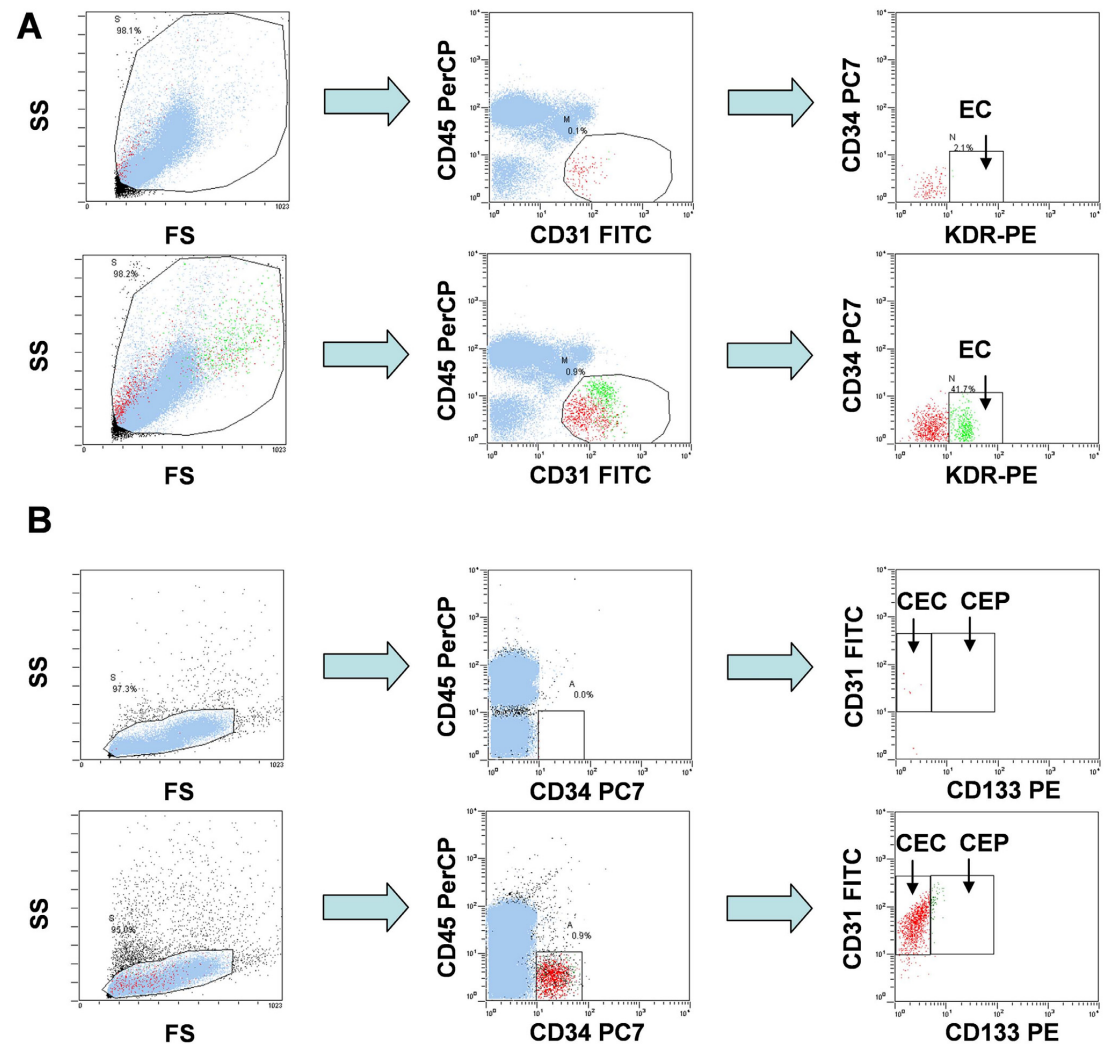
**Four-color flow cytometric analyses of endothelial cells within human mononuclear cells**. **A) **Staining for CD31, CD34, CD45 and KDR of PBMNC allows the detection of mature circulating endothelial cells. Top row: PBMNC sample of a healthy donor; lower row: autologous ECFC spiked into PBMNC from the respective healthy donor. **B **Staining for CD31, CD34, CD45 and CD133 of PBMNC allows the detection of circulating endothelial and progenitor cells. Top row: PBMNC sample of a healthy donor; lower row: cord-blood derived progenitors spiked into PBMNC from a healthy donor. Of note, only a small subset of cells displayed a CD34+ CD133 phenotype.

At first, the phenotypes of cord blood derived progenitor cells and ECFC was determined by flow cytometry (figure 2). As expected, ECFC were CD45^- ^CD31^+ ^(figure 2A). Further subtyping revealed that cells were CD34^-^KDR^+^. Moreover, cells were CD133^- ^and CD144^+ ^(data not shown). Due to the two-step isolation procedure cord blood progenitor cells displayed a CD45^-/low ^CD34^+ ^phenotype (figure 2B). The majority of CD34^+ ^cells displayed a CD31^+ ^CD133^- ^phenotype corresponding to hematopoietic precursor cells. In accordance with previous reports only a small subset of cells displayed a CD31^+ ^CD133^low ^phenotype (5–10% of the CD34+ cells)[9]. Therefore, only few cord blood-derived progenitor cells were detectable in the CEP window during the subsequent spiking experiments. Moreover, progenitor cells were KDR^- ^and CD144^- ^(data not shown).

### Detection of progenitors spiked into peripheral blood samples

**Figure 3:**
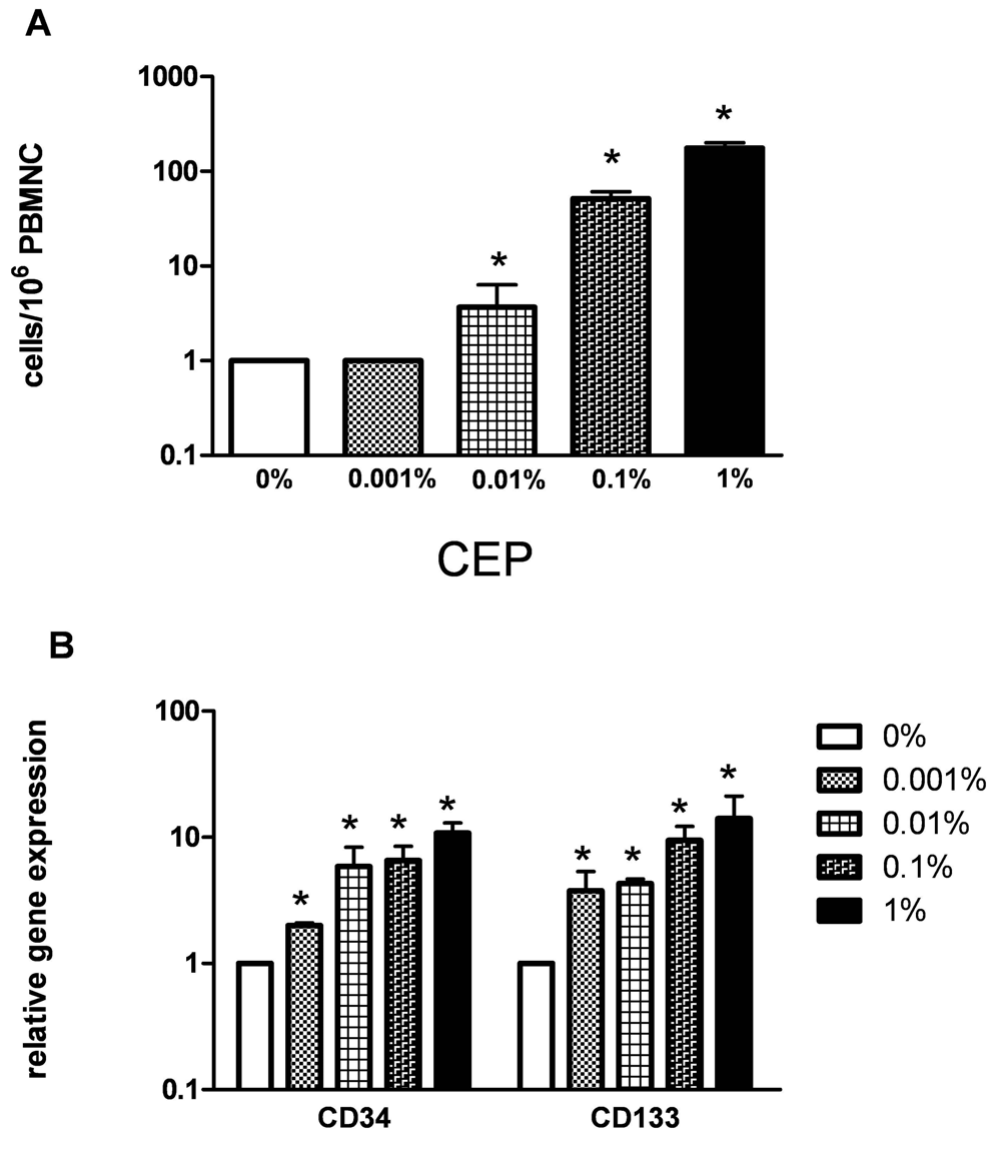
**Detection of progenitor cells spiked into peripheral blood samples**. A) Four-channel flow cytometric analysis of cord blood derived progenitor cells spiked into PBMNC (n = 5). Progenitors were spiked at frequencies ranging from 0.001 to 1% into PBMNC of a healthy donor. (* p < 0.05 compared to unspiked controls). B) Quantitative PCR analysis of CEP marker gene expression in peripheral blood samples containing cord blood derived progenitor cells spiked at varying frequencies (0.001–1%; five independent experiments). Expression of CD34 and CD133 was analyzed in all samples relative to unspiked PBMNC. (* p < 0.05 compared to unspiked controls).

In five independent experiments cord blood derived progenitors from different donors were spiked in triplicates into PBMNC from healthy volunteers at concentrations ranging from 0.001 to 1%. As depicted in figure 3A, flow cytometry allowed detection of progenitors with a CEP phenotype at concentrations ≥ 0.01% (mean: 4 cells/10^6 ^PBMNC vs 1 cell in unspiked controls, p = 0.017).

Quantification of both CD34 and CD133 gene transcripts proved to be a reliable approach for detecting spiked progenitor cells in PBMNC samples with 10-fold greater sensitivity than flow cytometry. At a frequency of only 0.001% progenitor cells were detectable by a 3.9-fold increased gene expression for CD133 (SD 2.2) and a 3.4-fold increase for CD34 (SD 2.3) in comparison to unspiked controls (p = 0.03; figure 3B). Although linear, the increase of CD34 and CD133 gene transcripts was not proportional to the number of cells spiked, possibly due to technical reasons (e.g., cell clumping) or cell death as progenitor cells are more fragile compared to mature cells. Due to their low expression on progenitors as described above, CD144 and KDR gene expression was not analyzed.

### Detection of ECFC spiked into peripheral blood samples

**Figure 4:**
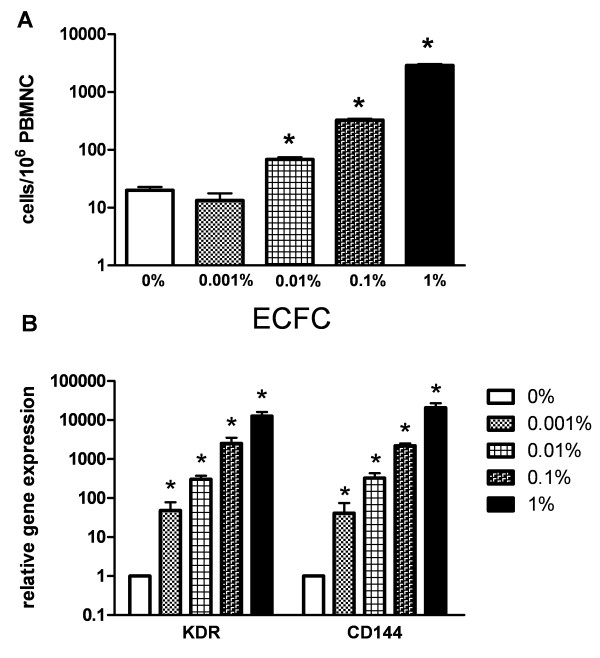
**Detection of ECFC in peripheral blood samples**. **A) **Four-channel flow cytometric analysis of autologous ECFC spiked into PBMNC of the respective donor (n = 5). ECFC were spiked at frequencies ranging from 0.001 to 1%. (* p < 0.05 compared to unspiked controls). **B) **Quantitative PCR analysis of ECFC marker gene expression in peripheral blood samples containing ECFC spiked at varying frequencies (0.001–1%; five independent experiments involving five different healthy donors). Expression of KDR and CD144 was analyzed in all samples relative to unspiked PBMNC. (* p < 0.05 compared to unspiked controls).

In five independent experiments freshly detached autologous ECFC were spiked into PBMNC samples of the respective donor at concentrations ranging from 0.001 to 1%. Using flow cytometry ECFC spiked into PBMNC samples were detectable at frequencies ≥ 0.01% (mean: 68 cells/10^6 ^PBMNC vs 20 cells in unspiked controls, p = 0.004; figure 4A).

QPCR was at least 10-fold more sensitive than flow cytometry for ECFC detection. Indeed, at a frequency of 0.001% relative gene expression of KDR was increased 48.3-fold (SD 52.1; p = 0.027) and that of CD144 40.8-fold (SD 58.5; p = 0.04) compared to unspiked controls (figure 4B). Due to their low expression level on ECFC as described above, CD34 and CD133 gene transcripts were not determined in this setting.

### Detection of CEC/CEP in peripheral blood of cancer patients and healthy volunteers

**Figure 5:**
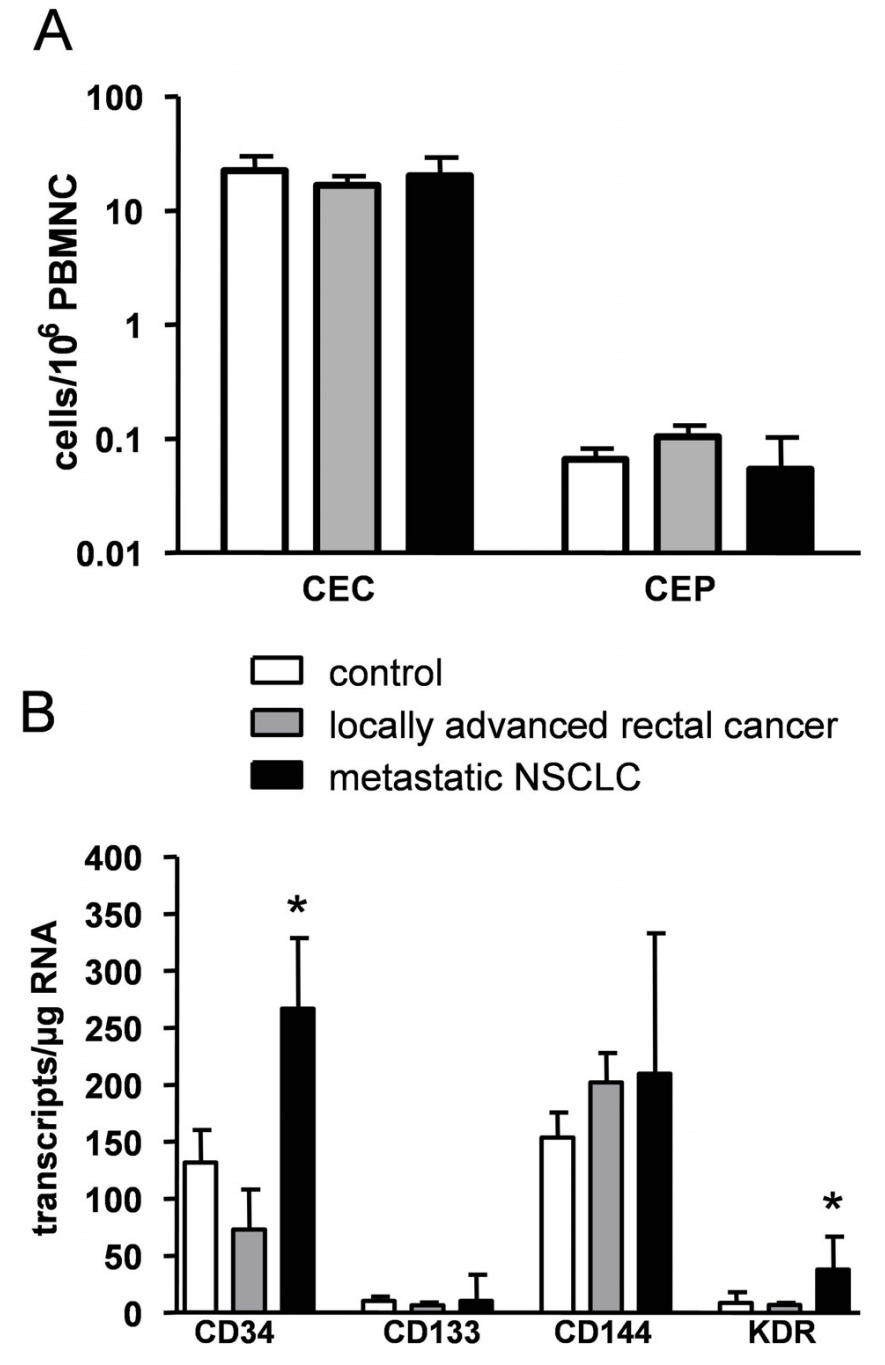
**Quantification of CEC and CEP in cancer patients and healthy controls**. The content of CEC and CEP was determined by flow cytometry and qPCR in peripheral blood of patients with newly-diagnosed rectal cancer patients (n = 20), metastatic NSCLC patients (n = 25) and healthy controls (n = 30). Statistics were performed using the Kruskal Wallis H test. (**A**) Applying flow cytometry no difference was found concerning CEC/CEP detection in the peripheral blood of cancer patients and healthy controls. (Note the logarithmic scale.) (**B**) In comparison to healthy controls gene expression of endothelial cell markers CD34 and KDR was significantly elevated in patients with metastatic NSCLC but not in patients with newly-diagnosed localized rectal cancer patients. (* p < 0.05; bars indicate mean +/- SD).

Applying 4-color flow cytometry no significant differences concerning CEC/CEP numbers were found between cancer patients and age-matched healthy controls (figure 5A). We then applied qPCR for quantifying gene transcripts of endothelial progenitor cell markers CD34, CD133 and endothelial cell markers KDR and CD144. Overall, gene transcripts for CD34 and CD144 were found abundantly expressed (i.e., >100 copies/μg RNA) in the total study cohort whereas only very few KDR or CD133 gene transcripts were detectable (figure 5B). Subgroup analysis showed no influence of gender, age or tumor stage on any of the markers studied. No significant elevation of any of the endothelial marker gene transcripts determined was found in locally advanced rectal cancer patients. In contrast, in the cohort of patients with metastatic NSCLC a significantly increased CD34 and KDR gene expression was found (p = 0.028 and p = 0.002, respectively).

## Discussion

Recently, CEC and CEP have been suggested as surrogate markers for angiogenesis and response to antiangiogenic therapy in cancer [10]. Flow cytometric rare event analysis, currently the most commonly applied method for CEC/CEP assessment, is technically demanding due to a high level of "background noise", i.e. false positive events due to autofluorescence, cell clumps and non-specific staining of other cell types such as monocytes, lymphocytes, non-lysed erythrocytes, aggregated platelets [11], dead cells or endothelial microparticles. Furthermore, technical aspects such as inadequate cleaning of the cytometer, blocking, washing and lysing procedures may impact the flow-cytometric analysis. Even with freshly drawn blood and standardized technical workup non-specific binding of fluorochrome-matched isotype controls may be observed in up to 0.5% of cells analyzed markedly exceeding the anticipated number of CEC and CEP within the PBMNC fraction (0.01% to 0.0001%) [12]. As a result, CEC/CEP measurements are virtually not comparable between different laboratories posing a major obstacle for the interpretation of the data published in the literature.

The detection limit of 0.001% CEC/CEP in PBMNC determined for qPCR in our spiking experiments is compatible with frequencies of CEC/CEP in humans reported in the literature [1] and well above the values determined by 4-color flow cytometry (i.e., 0.01%).

However, despite markedly improved sensitivity determined in our spiking experiments we found normal endothelial marker gene expression when we applied qPCR to samples from patients with newly diagnosed locally advanced rectal cancer. This may be due to the rather low tumor burden in this study cohort as overall angiogenic activity depends not only on the tumor type but also on tumor load. When we analysed blood samples from patients with a high tumor burden, i.e., metastatic NSCLC, we found significantly elevated gene expression levels of CD34 and KDR which may indicate an elevated number of CEC. Importantly, these markers are not specific for endothelial cells and might as well reflect circulating hematopoietic progenitors due to tumor-associated inflammatory stimuli, metastatic cells or platelet contamination. This represents the major limitation of the qPCR methodology as applied in our study: gene transcript quantification is carried out on total RNA derived from PBMNC. Thus and in contrast to flow cytometry, qPCR does not allow to identify distinct cell types through simultaneous assessment of multiple markers on one cell and the detection of CD34 and KDR gene expression alone in PBMNC is by no means proof for CEC/CEP. But currently there are no specific CEC/CEP markers available, crucial for a valid molecular detection assay. Enrichment procedures (e.g., immunomagnetic beads for CD146) are currently being studied to improve CEC detection limits achievable with flow cytometry. However, it remains to be determined whether these labor-intensive techniques can provide the purity and cell numbers required for proper flow cytometric CEC enumeration.

In conclusion qPCR is more sensitive, but less specific than 4-channel flow cytometry for the detection of CEC/CEP. Nevertheless, both methods failed to reliably detect an increase of CEC/CEP in tumor patients. However, despite significant improved detection limits by qPCR a single marker expressed specifically in CEP/CEC is hitherto missing. Such a marker would be crucial to achieve high specificity and to discriminate these rare cells from other cell populations of the peripheral blood. Thus, transcriptome analysis of sorted and functionally tested CEC/CEP might lead to the discovery of novel markers that can be used in real-time PCR-based assays.

## Methods

### Acquisition of blood samples

The study was carried out according to the regulations of the local ethics committee and Austrian Law. After having obtained informed consent, peripheral blood samples were drawn from patients with newly diagnosed, locally advanced rectal cancer (n = 20), metastatic non-small cell lung cancer (NSCLC; n = 25) and from age-mached healthy volunteers (n = 30). Women during the active menstrual phase were excluded from the study. Patients' characteristics are summarized in table 1.

**Table 1:** Patient characteristics

Parameters	**No. of patients**
Rectal cancer patients	20
UICC stage I	6 (30%)
UICC stage II	5 (25%)
UICC stage III	9 (45%)
median age (years)	64
female	6 (30%)
NSCLC patients	25
UICC stage IV	25 (100%)
median age (years)	65
female	10 (40%)

### Isolation of progenitor cells from cord blood

Human umbilical cord blood samples (n = 10) were obtained at birth from full-term newborns. Blood samples were collected in heparinized tubes and stored at 8°C no longer than 12 h before flow cytometric analysis and mRNA extraction, respectively. MNC were isolated by Ficoll density gradient centrifugation (Lymphoprep^®^, Nycomed, Norway). Progenitor cells were enriched by a two step immunomagnetic bead separation protocol by negative selection for CD45 and subsequent positive selection for CD34^+ ^(CD34 isolation kit, CD45 microbeads, Miltenyi Biotec). Progenitor cells were spiked into PBMNC of healthy volunteers at frequencies ranging from 0.001 to 1%.

### Generation of endothelial colony forming cells (ECFC) from peripheral blood

Autologous ECFC cultures were generated as described previously [7,13]. Briefly, PBMNC from five healthy donors were isolated by Ficoll density gradient centrifugation, resuspended in EGM-2 medium (Cambrex) and placed into a six-well-plate coated with type I collagen (from kangaroo, Sigma-Aldrich). After 24 h, non-adherent cells were removed by changing the medium. Autologous ECFC were spiked into PBMNC of the corresponding donor at frequencies ranging from 0.001 to 1%.

### Flow cytometry

Flow cytometric detection and enumeration of of CEC/CEP was carried out according to a recently published protocol [14]. Except for the anti-CD34 antibody monoclonal antibodies were chosen exactly as suggested by the authors. In brief, after Fc-blocking (Fc-receptor blocking antibody, Miltenyi Biotec) PBMNC were incubated in triplicates with antibodies specific for CD31-FITC (BD Pharmingen), CD34-PC7 (Beckman Coulter), CD45 PerCP (BD Pharmingen), CD133-PE (Miltenyi Biotec) or VEGF-R2 (KDR)-PE (R&D Systems). Appropriate fluorochrome-conjugated isotype-matched murine IgG antibodies (BD Pharmingen) were used as controls for each staining procedure. After incubation for 30 min at 4°C, cells were washed, resuspended in 300 mL PBS and analyzed in a Cytomics-FC-500 cytometer using the Cytomics RXP-Software (Beckman Coulter; figure 2). CEC were defined as CD31^+^/CD34^+^/CD45^-^/CD133^- ^and CEP were defined as CD31^+^/CD34^+^/CD133^+^/CD45^-/low ^cells. All experiments were carried out in triplicates with analysis of at least 5 × 10^5 ^cells per run.

### Quantitative PCR

RNA was purified by cell lysis of 5 × 10^5 ^PBMNC and nucleic acid extraction using of the RNeasy Kit (Qiagen). Extracted total RNA was transcribed into cDNA with oligo-dT- und hexanukleotide-random-primers and the AMV-Reverse Transcriptase (all Promega). For qPCR analysis in the 20 ng of each cDNA were used in triplicates. 5 μL Sybr-green Mix (Bio-Rad), and 10 pMol of each primer were mixed to the cDNA sample. Primer sequences and PCR conditions are listed in table 2. Instead of determining the ubiquitously expressed CD31 gene we assessed gene expression of the more specific endothelial marker CD144. Analysis was carried out in a Bio-Rad iCycler using the iCycler Software (Bio-Rad). Efficiency of the used primer pairs was determined by logarithmic dilutions of a highly concentrated cDNA template. Relative quantification and statistical data analysis of triplicates per sample was done according to the delta-Ct method described by Pfaffl et al. [15].

**Table 2:** Genes and primer sequences used for qPCR

**Gene**	Sequence	Tm	Assay range cycles	Primer efficiency
**EF1A **(EEF1A1)	For: 5-cacacggctcacattgca Rev:5-cacgaacagcaaagcgacc	86	12–30	98%
**CD34 **(HPCA1)	For: 5-tccagagacaaccttgaagc Rev: 5-cttcttaaactccgcacagc	85	18–32	100%
**CD133 **(PROM1)	For: 5-ttgcggtaaaactggctaag Rev: 5-tgggcttgtcataacaggat	81	20–36	100%
**KDR **(VEGFR2)	For: 5-gtggggattgacttcaactg Rev: 5-tgtgctgttcttcttggtca	85	17–35	98%
**CD144 **(CDH5)	For: 5-ttcatgacgtgaacgacaac Rev: 5-tccaccacgatctcatacct	89	17–35	84%

### External standards

For absolute quantification CD34, CD133, KDR and CD144 cDNAs were subcloned by the use of the PCR-Script cloning kit (Stratagene). Plasmid copies were calculated as follows: amount (copies/μL) = 6 × 10^23 ^(copies/mol) × concentration (g/μL)/MW (g/mol). Standard curves were generated by logarithmic dilutions of triplicates of the amplicon-containing plasmids in a complex matrix of COS-7 cDNA. These external standard curves were used to calculate copy numbers per μg total RNA in all PBMNC samples using the iCycler software (BioRad).

### Statistical analysis

Statistical analysis was performed with the GraphPad Prism 5 software for Windows. All tests of statistical significance were two-sided. Student's t-test was used for the analysis of spiking experiments. Kruskal Wallis H test was applied to study differences between healthy controls, rectal cancer patients and NSCLC patients.

## Competing interests

The authors declare that they have no competing interests.

## Authors' contributions

MS and GU designed and coordinated the study, provided funding, analyzed the data and wrote the manuscript. JK carried out cell isolation assays and cell culture. MZ and AA provided rectal cancer samples, corresponding clinical data and performed RNA isolation. MB carried out flow cytometric analyses and qPCR assays. EG and GG participated in the design of the study, critical discussion of study data and writing of the manuscript. All authors read and approved the final manuscript.
